# The inhibition of calpains ameliorates vascular restenosis through MMP2/TGF-β1 pathway

**DOI:** 10.1038/srep29975

**Published:** 2016-07-25

**Authors:** Lianghu Tang, Haifeng Pei, Yi Yang, Xiong Wang, Ting Wang, Erhe Gao, De Li, Yongjian Yang, Dachun Yang

**Affiliations:** 1 Department of Cardiology, Chengdu Military General Hospital, Chengdu 610083, China; 2 Department of Cardiology, The Affiliated Hospital of Southwest Medical University, Luzhou 64600, China; 3 Department of Cardiology, Xijing Hospital, Fourth Military Medical University, Xi’an 710032, China; 4 Center of Translational Medicine, Temple University School of Medicine, Philadelphia, PA 19140, USA

## Abstract

Restenosis limits the efficacy of vascular percutaneous intervention, in which vascular smooth muscle cell (VSMC) proliferation and activation of inflammation are two primary causal factors. Calpains influence VSMC proliferation and collagen synthesis. However, the roles of calpastatin and calpains in vascular restenosis remain unclear. Here, restenosis was induced by ligating the left carotid artery, and VSMCs were pretreated with platelet-derived growth factor (PDGF)-BB. Adenovirus vector carrying MMP2 sequence and specific small interfering RNA against calpain-1/2 were introduced. Finally, restenosis enhanced the expression of calpain-1/2, but reduced calpastatin content. In calpastatin transgenic mice, lumen narrowing was attenuated gradually and peaked on days 14–21. Cell proliferation and migration as well as collagen synthesis were inhibited in transgenic mice, and expression of calpain-1/2 and MMP2/transforming growth factor-β1 (TGF-β1). Consistently, in VSMCs pretreated with PDGF-BB, calpastatin induction and calpains inhibition suppressed the proliferation and migration of VSMCs and collagen synthesis, and reduced expression of calpain-1/2 and MMP2/TGF-β1. Moreover, simvastatin improved restenosis indicators by suppressing the HIF-1α/calpains/MMP2/TGF-β1 pathway. However, MMP2 supplementation eliminated the vascular protection of calpastatin induction and simvastatin. Collectively, calpains inhibition plays crucial roles in vascular restenosis by preventing neointimal hyperplasia at the early stage via suppression of the MMP2/TGF-β1 pathway.

In patients who receive percutaneous interventions of coronary, carotid and peripheral arteries, restenosis will lead to recurrent lumen narrowing^1^,^2^,^3^. Considering the rapidly increasing number of cardiovascular patients^4^, restenosis has become a significant clinical concern. There are many pathological features of in-stent restenosis that limit the efficacy of percutaneous intervention^5^, including dysfunctional endothelial cells, proliferation and migration of vascular smooth muscle cells (VSMCs), and activation of inflammation^6^. As the principal component of vascular walls, VSMCs play crucial roles in both the physiological functions of blood vessels and the formation atherosclerotic lesions^7^. As induced by cytokines and growth factors including platelet-derived growth factor (PDGF) during atherosclerosis and restenosis, abnormal VSMC proliferation and migration will give rise to obvious neointimal formation and severe vascular lumen loss^8^, in which PDGF initiates a multitude of biological effects by activating certain intracellular signal transduction pathways^9^. Thus, inhibition of VSMC proliferation and migration induced by PDGF may represent an important therapeutic intervention for restenosis after angioplasty.

As a growing family of cysteine proteinases whose activity depends on intracellular Ca^2+^ concentrations^10^, calpains perform important roles in basic physiological and pathological processes^11^,^12^. All calpain isoforms are located in the cytosol as inactive proenzymes with calpain-μ (or 1) and calpain-m (or 2) expressed ubiquitously. There are many processes involved in calpain activation, such as calcium influx, phospholipid binding, release of calpain from its inhibitor, binding of activator proteins, and phosphorylation^13^. PDGF is able to activate calpain-1/2 by increasing the intracellular Ca^2+^ concentration and activation of mitogen-activated protein kinase^14^,^15^. Furthermore, calpains mediate PDGF-induced collagen synthesis and VSMC proliferation^16^. It is well known that deregulated calpain activity can cause tissues damage in response to certain events such as myocardial infarct, stroke, and brain trauma^17^. Overactivation of calpain leads to pulmonary vascular remodelling induced by arterial hypertension^18^ and cardiovascular remodelling induced by angiotensin II^19^. Moreover, calpastatin serves as the major endogenous inhibitor of calpains^20^ via binding to calpains in the presence of Ca^2+^ 21,22. Thus, the expression level of calpastatin is likely to be an important factor in controlling calpain activity. Accumulating evidence suggests a modulatory role of the calpastatin/calpains pathway in cardiovascular remodelling^18^,^19^,^23^. However, the alterations of calpastatin and calpains under the conditions of in-stent restenosis are incompletely understood, regardless of the related biological functions and underlying mechanisms.

The neointimal formation in vascular restenosis may be associated with abnormal proliferation and migration of VSMCs and new collagen deposition. Belonging to a broad family of Zn^2+^-binding endopeptidases, matrix metalloproteinase-2 (MMP2) plays crucial roles in cell proliferation, migration, and collagen deposition^24^,^25^,^26^. With membrane type 1 matrix metalloproteinase (MT1MMP) and tissue inhibitor of metalloproteinase 2 (TIMP2) being the most potent activator and most important inhibitor of MMP2 activity, respectively, the ratio of MT1MMP/TIMP2 will finally determine the effects of MMP2^27^. Calpain-1 activation appears to be a pivotal event in MMP2 activation and synthesis of collagen I and III^28^,^29^,^30^. Calpain-2 can also increase MMP2 activity to promote glioblastoma cell invasion^31^. As a major profibrotic factor, transforming growth factor-β1 (TGF-β1) can be activated by increased MMP2 to induce collagen production in the central arterial wall^32^. Moreover, a neutralizing antibody against TGF-β1 can block the effects of MMP2 on collagen synthesis^26^. Notably, Leloup *et al*.^33^ have revealed that calpain-2 participates in TGF-β1-mediated migration of myoblasts. Ma *et al*.^16^ also show that calpain can increase TGF-β1 expression to mediate PDGF-induced collagen synthesis and the proliferation of artery smooth muscle cells in pulmonary hypertension. However, whether MMP2/TGF-β1 signalling mediates the biological functions of calpastatin and calpain in vascular restenosis needs further research.

The aims of the present study were to determine (i) whether expression of calpastatin and calpains is altered in vascular restenosis; (ii) whether calpastatin induction prevents vascular restenosis and, if so; (iii) to identify whether MMP2/TGF-β1 signalling-mediated proliferation and migration of VSMCs and synthesis of collagen influence the abovementioned effects; (iv) whether the abovementioned pathway participates in the biological actions of statins in restenosis.

## Methods

### Animal preparation

Calpastatin transgenic (TG) mice [C57BL/6J background, established with human calpastatin cDNA cloned into the pDown-calpastatin vector (Cyagen Biosciences Inc., Guangzhou, China)] and wild-type (WT) littermates were established and characterised as reported previously^23^. All mice were fed standard laboratory animal chow with free access to tap water, and were housed in a temperature- and humidity-controlled room with a 12/12-hour light-dark cycle. All experiments were performed in accordance with the National Institutes of Health Guidelines on the Use of Laboratory Animals and were approved by the Institutional Animal Care and Use Committee of Chengdu Military General Hospital.

### Protocol for carotid restenosis induction

Mice were anesthetized with pentobarbital sodium (40 mg/kg, i.p., Chengdu, China). The left carotid artery was dissected through a midline cervical incision. The left carotid artery just proximal to the bifurcation was then ligated with silk (6-0), whereas the right carotid artery was used as an internal control^34^. After 0, 1, 2, 3, and 4 weeks, mice were anaesthetized and sacrificed to harvest their carotid arteries.

### Culture of neonatal murine VSMCs

VSMCs were isolated from digested aortas. The cells were cultured at 37 °C with 5% CO_2_ in Dulbecco’s modified Eagle’s medium (DMEM) supplemented with Hepes buffer, 100 U/ml penicillin, 0.1 mg/ml streptomycin, glutamine, and 10% fetal calf serum (Invitrogen). VSMCs were characterized by immunostaining with a monoclonal antibody against α-actin (1:80, Abcam). All experiments were performed using confluent passage 2 or 3 cells after 24 hours of serum starvation.

### Gene silencing of calpain-1/2

Small interfering RNA (siRNA) was introduced to silence calpain expression in VSMCs. The specific siRNAs against calpain-1 and -2 were 5′-AAGCTAGTGTTCGTGCACTCT-3′ and 5′-AAACCAGAGCTTCCAGGAAAA-3′, respectively. The control sequence was 5′-AACGTACGCGGAATACTTCGA-3′. All siRNAs were synthesized by Qiagen (Chatsworth, CA, USA) and transfected into VSMCs using Qiagen RNAiFest transfection reagent as reported previously^35^. At 72 hours after transfection, protein expression of calpain-1/2 was evaluated in VSMCs.

### Adenoviral vector carrying MMP2

The *MMP2* gene was subcloned into an adenovirus expression vector, which also encoded green fluorescence protein (GFP), to construct an overexpression vector carrying *MMP2* (GenePharma, China)^36^. The recombinant plasmid was transfected into 293T cells to generate adenoviruses carrying GFP (AdGFP) or the mature MMP2 sequence (AdMMP2). Then, TG mice received a single intravenous injection (≈10^8^ pfu) of AdGFP or AdMMP2 via their tail vein at the time of ligation and at 7 days after ligation. In addition, VSMCs (grown to 70–80% confluence) from TG mice were transduced with AdGFP or AdMMP2 (≈3 pfu/cell). After 72 hours, VSMCs were collected for further experiments.

### Cell proliferation assay

PDGF-BB (R&D) was dissolved in 0.05% dimethyl sulfoxide and diluted further in serum-free DMEM. The effect of PDGF on VSMC proliferation was assessed with a cell counting kit-8 (CCK8) assay. In brief, VSMCs were seeded in 96-well plates (1 × 10^4^ cells per well) in the presence or absence of PDGF-BB (10 ng/ml) and cultured for 24 hours. CCK8 (0.2 mg/mL) was then added to each well, followed by incubation for 4 hours at 37 °C. Cell proliferation was assessed by measuring the absorbance at 450 nm using a microplate reader.

### Cell migration assay

For the transwell assay, we used 24-well modified Boyden chambers containing fibronectin-coated polycarbonate membranes (8-μm pore-size, BD Bioscience). Briefly, the lower wells of the chamber were filled with phenol red-free DMEM with or without PDGF-BB (10 ng/mL). The filters were coated with fibronectin (50 mg/ml) and fixed on top of the bottom wells. VSMCs (1 × 10^5^ cells per well) were allowed to migrate for 6 hours, and non-migrated cells were removed from the upper side of the membrane with cotton swabs. Cells on the lower side of the membrane were stained with 4,6-diamidino-2-phenylindole and counted in five randomly selected fields per well under a fluorescence microscope (Nikon, Japan). Data are presented as numbers of migrated cells per field^37^.

### Wound healing assay

For the scratch wound healing assay, VSMCs were seeded in 6-well plates (1.5 × 10^5^ cells per well) and grown to confluence. After 24 hours of serum deprivation, the cells were incubated for 24 hours in the presence or absence of PDGF-BB (10 ng/mL). Using direct microscopic visualization, wound closure rates were followed with a reference point in the field of the wound at the bottom of the plate. The procedure permitted photographing of an identical spot each time. At 24 hours after injury, the remaining cell-free area was determined by microphotography^37^.

### Immunohistochemical staining

After perfusion with saline, carotid arteries were fixed with 4% paraformaldehyde and embedded in paraffin. Thin sections (4–5 μm) were stained with haematoxylin & eosin (HE). The areas of intima and media of the carotid artery were measured using Image-Pro Plus software. Cell proliferation was assessed by immunohistochemical staining of proliferation cell nuclear antigen (PCNA) with the corresponding antibody (1:200, Abcam). Protein expression was evaluated by immunohistochemical staining using antibodies against calpastatin, calpain-1, calpain-2 (1:100, Abcam), MMP2, MT1MMP, TIMP2, and collagen I (1:100, Boster).

### Quantification of mRNA

According to the manufacturer’s instructions, total RNAs from carotid arteries or VSMCs were extracted using TRIzol (Invitrogen). Total RNA (2 μg) was reverse transcribed into cDNA. mRNA levels were quantified by quantitative real-time polymerase chain reaction (qRT-PCR) using SYBRGreen Master Mix (Takara). Samples were amplified using the Mastercycler ep Realplex2 System (Eppendorf, Hamburg, Germany). 18s rRNA served as an internal control for total cDNA content. Primer sequences were as follows: mouse calpastatin-F: 5′-ACGTAAACGGCCACAAGTTC-3′ and mouse calpastatin-R: 5′-GATCTTG AAGTTCACCTTGATGC-3′; human calpastatin-F: 5′-ACGTAAACGGCCACAAGTTC-3′ and human calpastatin-R: 5′-GATCTTGAAGTTCACCTTGATGC-3′; calpain-1-F: 5′-ACCA CATTTTACGAGGGCAC-3′ and calpain-1-R: 5′-GGATCTTGAACTGGGGGTTT-3′; calpain-2-F: 5′-CCCCAGTTCATTATTGGAGG-3′ and calpain-2-R: 5′-AAGCTCTGATCTGGAGGCAC-3′; MMP2-F: 5′-GCACTCTGGAGCGAGGATAC-3′ and MMP2-R: 5′-GCCCTC CTAAGCCAGTCTCT-3′; TGF-β1-F: 5′-GGCGATACCTCAGCAACCG-3′ and TGF-β1-R: 5′-CTAAGGCGAAAGCCCTCAAT-3′; collagen I-F: 5′-GTGGTAACGATGGTGCTGTCG-3′ and collagen I-R: 5′-CCAGCAACACCATCAGCACC-3′; collagen III-F: 5′-TGCAGGACCT AGAGGAGTAGC-3′ and collagen III-R: 5′-GTCCAGCTCCACCTCTAAGC-3′; 18s rRNA-F: 5′-CGCGGTTCTATTTTGTTGGTTT-3′ and 18s rRNA-R: 5′-GCGCCGGTCCAAGAATTT-3′. All samples were run in triplicate and averaged.

### Western blotting

VSMCs were lysed in RIPA buffer. Protein concentrations were quantified with protein assay reagent (Bio-Rad, Hercules, CA, USA). Equal amounts of protein were loaded and separated by 10% sodium dodecyl sulfate-polyacrylamide gel electrophoresis and then transferred onto polyvinylidene fluoride membranes (Bio-Rad). The membranes were incubated overnight with primary antibodies against HIF-1α (1:1000, Abcam), calpastatin, TGF-β1 (1:2000, Abcam), calpain-1, calpain-2, a-II spectrin (1:3000, Abcam), MMP2, TIMP2 (1:400, Boster), MT1MMP (1:200, Boster), and glyceraldehyde-3-phosphate dehydrogenase (GAPDH, 1:1000, Cell Signaling Technology). At room temperature, membranes were incubated with corresponding secondary antibodies for 1 hour. Quantitative analysis was performed with Image J software (NIH). All samples were run in triplicate and averaged.

### Statistical analysis

All values are presented as the mean ± standard error of the mean (SEM). Data (except western blot densities) were subjected to analysis of variance followed by Bonferroni correction for the post-hoc *t*-test. Western blot densities were analysed with the Kruskal–Wallis test followed by the Dunn post-hoc test. *P* ≤ 0.05 was considered to be statistically significant.

## Results

### Calpastatin induction attenuates vascular restenosis

**Figure 1:**
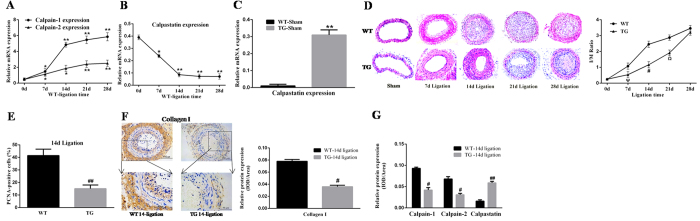
Alterations of calpain-1, calpain-2, and calpastatin expression in carotid restenosis with calpastatin induction attenuating restenosis. (**A,B**) mRNA levels of calpain-1, calpain-2, and mouse calpastatin were assessed by quantitative real-time polymerase chain reaction (qRT-PCR). (**C**) mRNA levels of human calpastatin were assessed by qRT-PCR. (**D**) The extent of carotid restenosis was assessed by haematoxylin & eosin (HE) staining. Representative images are shown in the left panel. The results were analysed by I/M ratio in the right panel. (**E**) Cell proliferation was assessed by immunohistochemical staining of PCNA-positive cells in carotid restenosis at 14 days after ligation. (**F**) Collagen synthesis was determined by immunohistochemical staining of collagen I at 14 days after ligation. Representative images are shown in the left panel. The results were automatically counted and calculated by Image-Pro Plus software as shown in the right panel. (**G**) Expression of calpain-1, calpain-2, and calpastatin was assessed by immunohistochemical staining at 14 days after ligation. The results were analysed by the IOD/area. WT, wild-type; TG, calpastatin transgene; I/M, intima/media; PCNA, proliferating cell nuclear antigen; IOD, integral optical density. Presented values are means ± SEM. N = 6–8/group. ^*^P < 0.05, ^**^P < 0.01 vs. WT-Sham (0d); ^#^*P* < 0.05, ^##^*P* < 0.01 vs. WT-14d ligation; ^Ψ^*P* < 0.05 vs. WT-7d ligation; ^Ω^*P* < 0.05 vs. WT-21d ligation.

To elucidate the specific functions of calpastatin and calpains in restenosis induced by carotid artery ligation, we performed mRNA analyses of calpain-1/2 and calpastatin, which might undergo changes, from the onset of ligation to day 28. As a result, there was a significant increase of calpain-1/2 expression in the injured carotid artery with the development of restenosis (Fig. 1A). In contrast, as a specific endogenous calpain inhibitor, mouse calpastatin expression was inhibited gradually (Fig. 1B). Collectively, these data indicate that the functional balance of calpastatin and calpains may be disrupted in vascular restenosis. To test this hypothesis, we established TG mice that constitutively expressed high levels of human calpastatin (*p* < 0.01, Fig. 1C). Compared with WT mice, lumen narrowing in TG mice was ameliorated gradually and peaked on days 14–21 as measured by HE staining and the intima/media ratio (*p* < 0.05, Fig. 1D). Based on these observations, we further explored vascular injury at 14 days after ligation. PCNA-positive cells, an important index of cell proliferation, showed a declining tendency (*p* < 0.01, Fig. 1E; Supplemental Fig. 1A). Collagen I was successfully suppressed by calpastatin overexpression (*p* < 0.05, Fig. 1F). Moreover, in ligated TG mice, expression of calpain-1/2 was obviously inhibited (*p* < 0.05, Fig. 1G; Supplemental Fig. 1B,C), whereas calpastatin expression was elevated significantly (*p* < 0.01, Fig. 1G; Supplemental Fig. 1D). Taken together, these results suggest that calpastatin induction can successfully attenuate vascular restenosis by suppressing cell proliferation and collagen synthesis at the early stage, which is related to inhibition of calpain-1/2.

### Vascular protection conferred by calpastatin induction depends on negative regulation of MMP2/TGF-β1 signalling

**Figure 2:**
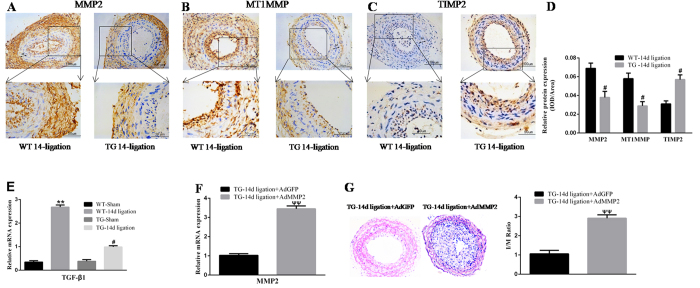
Calpastatin overexpression inhibits expression of MMP2/TGF-β1 in carotid restenosis, and MMP2 supplementation reverses the protective effect of calpastatin induction. (**A–C**) Expression of MMP2, MT1MMP, and TIMP2 was determined by immunohistochemical staining in carotid restenosis at 14 days after ligation. Representative images are shown. (**D**) Quantification of MMP2, MT1MMP, and TIMP2 by the IOD/area. (**E,F**) mRNA levels of MMP2/TGF-β1 were determined by qRT-PCR. (**G**) The extent of carotid restenosis was assessed by HE staining. MMP2, matrix metalloproteinase 2; MT1MMP, membrane type matrix metalloproteinase-1; TIMP2, tissue inhibitor of matrix metalloproteinase-2; TGF-β 1, transforming growth factor-β 1; IOD, integral optical density; AdGFP, adenovirus vector encoding green fluorescence protein; AdMMP2, adenovirus vector carrying MMP2 sequence. Presented values are means ± SEM. N = 6–8/group. ^**^P < 0.01 vs. WT-Sham; ^#^*P* < 0.05 vs. WT-14d ligation; ^ΨΨ^*P* < 0.01 vs. TG-14d ligation + AdGFP.

Substantial evidence has indicated that MMP2/TGF-β1 signalling might participate in cell proliferation and collagen synthesis. To identify the role of MMP2/TGF-β1 signalling in vascular restenosis, we detected the expression of these molecules in the injured carotid artery of TG mice. As a result, calpastatin overexpression markedly decreased the expression of MMP2, and the ratio of MT1MMP/TIMP2 showed a similar pattern compared with the WT-14d ligation group (*p* < 0.05, Fig. 2A–D). In addition, qRT-PCR analysis revealed a remarkable decline of TGF-β1 in TG mice (*p* < 0.05, Fig. 2E). Moreover, we administrated TG mice with an adenoviral vector carrying MMP2 through their tail vein. As a result, there was an obvious increase of MMP2 expression in the ligated carotid artery of TG mice (*p* < 0.01, Fig. 2F), which successfully reversed the protective effects of calpastatin induction against restenosis (*p* < 0.01, Fig. 2G). These combined data show that MMP2/TGF-β1 signalling is causally linked to the specific role of calpastatin in vascular restenosis.

### Calpastatin induction attenuates PDGF-induced proliferation and migration of VSMCs and collagen synthesis through inhibition of the MMP2/TGF-β1 pathway

**Figure 3:**
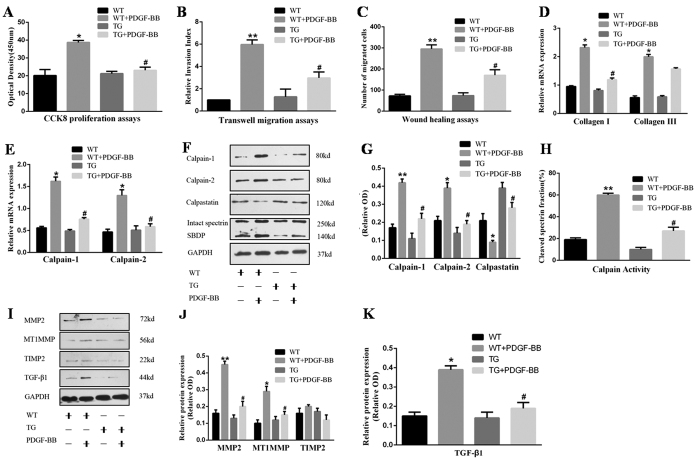
Calpastatin induction suppresses PDGF-induced proliferation and migration of VSMCs and collagen I synthesis via inhibition of the MMP2/TGF-β1 pathway mediated by calpain-1/2. (**A**) Cell proliferation was measured by cell counting kit-8 (CCK-8) assays. (**B**) Non-directional cell migration was measured by transwell migration assays. (**C**) Directional migration was measured by scratch wound healing assays. (**D,E**) mRNA levels of collagen I, collagen III, calpain-1, and calpain-2 were determined by qRT-PCR. (**F,I**) Representative images of western blots. (**G,J,K**) Densitometric analyses of calpastatin, calpain-1/2, MMP2, MTIMMP, TIMP2, and TGF-β1. (**H**) Calpain activity was determined by measurement of the fraction of calpain-specific SBDPs, which was calculated by dividing the cleaved spectrin density (145 and 150 kDa) by the total spectrin density (145, 150, and 250 kD). VSMCs, vascular smooth muscle cells; PDGF, platelet-derived growth factor; SBDPs, spectrin breakdown products; MMP2, matrix metalloproteinase 2; MT1MMP, membrane-type matrix metalloproteinase-1; TIMP2, tissue inhibitor of matrix metalloproteinase-2; TGF-β 1, transforming growth factor-β 1; OD, Optical density. Presented values are means ± SEM. N = 6–8/group. ^*^P < 0.05, ^**^P < 0.01 vs. WT; ^#^*P* < 0.05 vs. WT + PDGF-BB.

To identify the underlying mechanism of vascular restenosis, we isolated and cultured neonatal murine VSMCs (Supplemental Fig. 2A). The cells were then applied to CCK8, transwell, scratch wound healing, and qRT-PCR assays. We found that PDGF-BB markedly enhanced the proliferation and migration of VSMCs as well as collagen I and III synthesis, all of which were reversed by calpastatin overexpression (Fig. 3A–D; Supplemental Fig. 2B–D). In addition, the high expression of calpain-1/2 induced by PDGF-BB was mitigated by calpastatin induction as measured by qRT-PCR and western blotting (*p* < 0.05, Fig. 3E–G). Moreover, we determined the fraction expression of calpain-specific spectrin breakdown products (SBDPs), which indicated calpain activity. Notably, calpastatin induction obviously inhibited the activities of calpains (*p* < 0.05, Fig. 3F,H). Based on these observations, it is reasonable to assume that overexpression of calpastatin suppresses PDGF-induced expression of MMP2/TGF-β1 in cultured VSMCs. Finally, western blotting revealed that the increases in expression of MMP2/TGF-β 1 induced by PDGF-BB were reversed by calpastatin induction (*p* < 0.05, Fig. 3I–K). Therefore, these findings confirm that forced calpastatin expression in VSMCs ameliorates restenosis by inhibition of MMP2/TGF-β1 signalling.

### Calpains inhibition alleviates PDGF-induced proliferation and migration of VSMCs and collagen synthesis via suppression of the MMP2/TGF-β1 pathway

**Figure 4:**
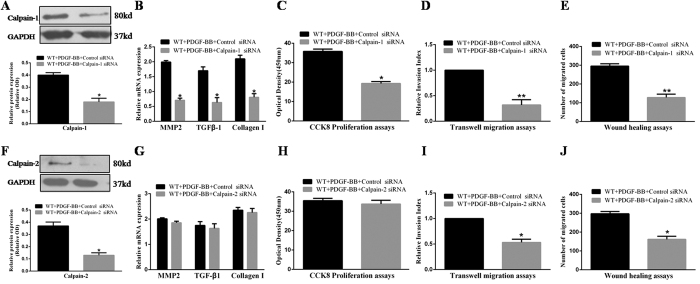
Calpains inhibition by siRNA attenuates PDGF-induced proliferation and migration of VSMCs and collagen synthesis by inhibition of the MMP2/TGF-β1 pathway. (**A,F**) Expression of calpain-1/2 was determined by western blotting. (**B,G**) mRNA levels of MMP2, TGF-β1, and collagen I were evaluated by qRT-PCR. (**C,H**) Cell proliferation was measured by CCK-8 assays. (**D,I**) Non-directional cell migration was measured by transwell migration assays. (**E,J**) Directional cell migration was measured by scratch wound healing assays. VSMCs, vascular smooth muscle cells; PDGF, platelet-derived growth factor; MMP2, matrix metalloproteinase 2; TGF-β1, transforming growth factor-β 1; siRNA, small interfering RNA; OD, optical density. Presented values are means ± SEM. N = 6–8/group. ^*^P < 0.05, ^**^P < 0.01 vs. WT + PDGF-BB + Control siRNA.

To identify the specific role of calpain-1/2 in vascular restenosis, we pretreated VSMCs with specific siRNAs against calpain-1 or -2. After 72 hours of transfection, expression of calpain-1/2 was inhibited (*p* < 0.05, Fig. 4A,F). In CCK8, transwell, scratch wound healing, and qRT-PCR assays, we found that calpain-1 inhibition largely attenuated PDGF-BB-induced proliferation and migration of VSMCs and collagen I synthesis, as well as expression of MMP2/TGF-β1 (Fig. 4B–E). In contrast, calpain-2 inhibition only ameliorated PDGF-BB-induced migration of VSMCs as evidenced by transwell and scratch wound healing assays (Fig. 4I,J). These results suggest that calpain-1 is the major molecule in the development of vascular restenosis by influencing all aspects, whereas calpain-2 serves as an auxiliary element in restenosis by only enhancing VSMC migration.

### MMP2 overexpression exacerbates PDGF-induced proliferation and migration of VSMCs

**Figure 5:**
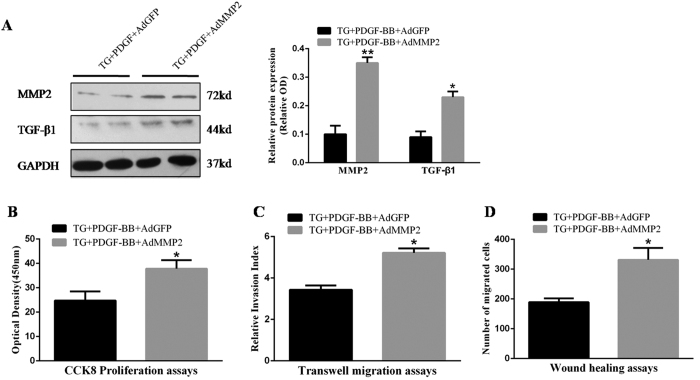
MMP2 overexpression reverses the protective effect of calpastatin induction in VSMCs pretreated with PDGF-BB. **(A)** Expression of MMP2 and TGF-β1 was determined by western blotting. **(B)** Cell proliferation was measured by CCK-8 assays. **(C)** Non-directional cell migration was measured by transwell migration assays. **(D)** Directional cell migration was measured by scratch wound healing assays. VSMCs, vascular smooth muscle cells; PDGF, platelet-derived growth factor; MMP2, matrix metalloproteinase 2; TGF-β 1, transforming growth factor-β 1; AdGFP, adenovirus vector encoding green fluorescent protein; AdMMP2, adenovirus vector carrying *MMP2*; OD, optical density. Presented values are means ± SEM. N = 6–8/group. ^*^P < 0.05, vs. WT + PDGF-BB + AdGFP.

To elucidate the causal role of MMP2 in the protective effects of calpastatin against restenosis, we pretreated TG VSMCs with an adenoviral vector carrying *MMP2*. We found an apparent increase in MMP2 expression (*p* < 0.01, Fig. 5A), along with increased TGF-β1 expression (*p* < 0.05, Fig. 5A). CCK8, transwell, and scratch wound healing assays showed that MMP2 overexpression significantly reversed the inhibitive effects of calpastatin on the proliferation and migration of VSMCs (*p* < 0.05, Fig. 5B–D). Therefore, these data indicated that MMP2/TGF-β1 indeed serves as downstream signalling of calpastatin and calpains in vascular restenosis.

### Simvastatin alleviates vascular restenosis via inhibition of the HIF-1α/calpains/MMP2/TGF-β1 pathway

**Figure 6:**
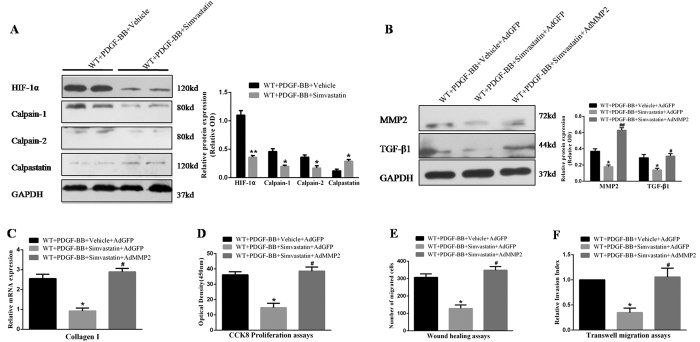
Simvastatin attenuates PDGF-induced proliferation and migration of VSMCs and collagen I synthesis, possibly by inhibition of the HIF-1α/calpains/MMP2/TGF-β1 pathway. (**A,B**) Expression of calpastatin, calpain-1/-2, HIF-1α, MMP2, and TGF-β1 was determined by western blotting. (**C**) The mRNA level of collagen I was determined by qRT-PCR. (**D**) Cell proliferation was measured by CCK-8 assays. (**E**) Non-directional cell migration was measured by transwell migration assays. (**F**) Directional cell migration was measured by scratch wound healing assays. VSMCs, vascular smooth muscle cells; PDGF, platelet-derived growth factor; MMP2, matrix metalloproteinase 2; TGF-β 1, transforming growth factor-β 1; AdGFP, adenovirus vector encoding green fluorescent protein; AdMMP2, adenovirus vector carrying *MMP2*; OD, optical density. Presented values are means ± SEM. N = 6–8/group. ^*^P < 0.05, ^**^P < 0.01 vs. WT + PDGF-BB + Vehicle or WT + PDGF-BB + Vehicle + AdGFP; ^#^*P* < 0.05, ^##^*P* < 0.01 vs. WT + PDGF-BB + Simvastatin + AdGFP.

Simvastatin has been shown to reduce inflammatory responses in vascular diseases. Thus, we investigated whether simvastatin alleviates vascular restenosis and analysed the regulation of simvastatin in the calpastatin/calpains system. As shown in Fig. 6A, simvastatin significantly reduced the expression of calpain-1/2 (*p* < 0.05) and HIF-1α (*p* < 0.01), and obviously increased the expression of calpastatin (*p* < 0.05). Moreover, simvastatin markedly suppressed the expression of MMP2 and TGF-β1 (*p* < 0.05, Fig. 6B), which was reversed by administration of the adenoviral vector carrying *MMP2*. Most importantly, the application of simvastatin significantly inhibited PDGF-induced synthesis of collagen I and the proliferation and migration of VSMCs, but not in the presence of AdMMP2 (*p* < 0.05, Fig. 6C–F). Consequently, these observations reveal that simvastatin alleviates restenosis by inhibition of the HIF-1α/calpains/MMP2/TGF-β1 pathway.

## Discussion

The present study provides a deep insight into the protective functions of calpastatin and calpains in vascular restenosis. First, we found disrupted expression of calpastatin and calpains in vascular restenosis, whereas calpastatin induction and calpains inhibition attenuated restenosis by inhibiting the proliferation and migration of VSMCs as well as collagen synthesis at the early stage. Second, we provided valid evidence indicating that MMP2/TGF-β1 signalling was negatively regulated by the calpastatin/calpains pathway to affect vascular restenosis. Third, we revealed that statins were able to prevent restenosis partly via inhibition of the HIF-1α/calpains/MMP2/TGF-β1 pathway. To the best of our knowledge, this is the first study employing TG mice to investigate the specific role of the calpastatin/calpains pathway in vascular restenosis.

Vascular percutaneous intervention is the main therapeutic strategy for atherosclerosis. However, the high incidence of vascular restenosis limits its clinical efficacy, and the detailed underlying mechanisms responsible for the occurrence and development of restenosis have not yet been fully elucidated yet^38^,^39^. Increasing evidence suggests that calpains play an important role in cell differentiation, proliferation and migration^40^,^41^,^42^. Recently, uncontrolled activation of calpain has been found to contribute to the pathogenesis of myocardial reperfusion injury, cardiac hypertrophy, myocardial stunning, and cardiac ischemia^43^. In pulmonary hypertension, Kovacs *et al*.^18^ showed that calpain modulates pulmonary vascular remodelling. In parallel, Ma *et al*.^16^ also revealed that calpain mediates PDGF-induced collagen synthesis and VSMC proliferation. Moreover, Letavernier *et al*.^19^ claimed that calpastatin overexpression can serve as an endogenous inhibitor to blunt calpain activation, and calpastatin TG mice display an obvious improvement in angiotensin II-induced left ventricle hypertrophy. However, to date, the specific function of the calpastatin/calpains pathway in vascular restenosis remains poorly defined. Here, through establishing mouse models of vascular restenosis by ligation of the left carotid artery, we found that the calpastatin/calpains pathway is negatively regulated in vascular restenosis, indicating its important role in restenosis. Next, using TG mice, we directly observed the protective effects of calpastatin/calpains signalling in vasculature. Data from the present study indicated that calpastatin gene transfer successfully attenuated vascular restenosis by suppressing cell proliferation and collagen synthesis at the earlier stage. Most importantly, the expression and activities of calpain-1/2 were largely inhibited in ligated TG mice. It has been reported that calpastatin overexpression reduces reactive oxygen species (ROS) production and peroxynitrite formation in diabetic mice^44^, and ROS overload is able to enhance the expression and activity of calpains^45^,^46^,^47^. Thus, calpastatin induction may not only directly prevent calpains activation, but also indirectly inhibit calpains expression by lessening ROS generation. These results reveal an important clinical significance in the treatment of in-stent restenosis by targeting calpastatin. To further elucidate whether calpain-1/2 play equal roles in restenosis, we introduced specific siRNAs against calpain-1/2 into VSMCs. As a result, we found that calpain-1 served as the major molecule to induce restenosis processes by enhancing the proliferation and migration of VSMCs and increasing collagen synthesis. In contrast, calpain-2 only played an auxiliary role in restenosis by enhancing VSMC migration. Thus, calpain-1 may be developed as an effective therapeutic target for restenosis patients.

To elucidate the underlying pathway responsible for the abovementioned alterations, we assessed expression changes of MMP2 and TGF-β1 that are pivotal in arterial restenosis after vascular intervention by influencing matrix deposition and cell proliferation^48^,^49^. It has been demonstrated that calpain-1 mediates MMP2 expression to enhance age-associated VSMC migration and collage deposition^28^. Furthermore, calpain-2 has been reported to increase MMP2 activity, resulting in enhanced glioblastoma cell invasion^31^. Moreover, Wang *et al*. have reported activation of TGF-β1 by increasing MMP2 to induce collagen production in the central arterial wall^32^. Here, we determined the expression of MMP2/TGF-β1 signalling by the application of immunohistochemistry and PCR in TG mice. The results from our study indicated that calpastatin overexpression markedly suppressed the expression of MMP2/TGF-β1 in the injured carotid artery. In addition, the finally decreased quantity of MMP2 was attributed to the reduction of MT1MMP expression and enhancement of TIMP2 expression. These findings revealed a close link between MMP2/TGF-β1 signalling and the pathogenesis of vascular restenosis at the downstream of the calpastatin/calpains pathway. To ascertain the specific location of calpastatin/calpains signalling, we cultured neonatal murine VSMCs that were distinguished by immunohistochemical staining of α-actin. Then, VSMCs were treated with PDGF-BB to establish an *in vitro* restenosis model. Lastly, our study demonstrated that overexpression of calpastatin attenuated PDGF-induced proliferation and migration of VSMCs and collagen synthesis in VSMCs. Moreover, calpastatin induction lessened the expression of calpain-1, calpain-2, MMP2, and TGF-β1 in parallel with the results obtained from the animal experiments. Most importantly, MMP2 supplementation successfully reversed the vascular protection of calpastatin induction, as evidenced by the *in vivo* and *in vitro* experiments. Taken together, these observations led to the reasonable deduction that inhibiting the expression of MMP2/TGF-β1 by calpastatin induction or calpains inhibition may be an effective method to prevent vascular restenosis as early as possible.

To test our hypothesis, we used statins that are widely used to lower plasma low-density lipoprotein cholesterol and play a clear role in the primary prevention of cardiovascular disease mortality and major events^50^. In addition to their roles in reducing cholesterol, statins may have additional anti-atherogenic effects, such as improving endothelial function, attenuating vascular and myocardial remodelling, stabilising atherosclerotic plaques, and inhibiting vascular inflammation^51^. In the present study, we found that simvastatin positively regulated calpastatin expression and obviously inhibited expression of HIF-1α, calpains, MMP2, and TGF-β1. These results suggest that statins can alleviate vascular restenosis by inhibiting the HIF-1α/calpains/MMP2/TGF-β1 pathway. Consistent with our findings, statins have been reported to enhance ubiquitin/proteasome-dependent degradation of HIF-1α^52^, and HIF-1α reduction will lead to a significant decrease in the expression and activity of calpains^53^. Moreover, simvastatin successfully attenuated PDGF-induced proliferation and migration of VSMCs as well as collagen synthesis, which was reversed by MMP2 overexpression. Thus, calpastatin/calpains signalling may be developed as a promising therapeutic target for vascular restenosis in the future. Further study is needed to assess the clinical efficacy of the calpastatin/calpains pathway, focusing on drugs that increase calpastatin expression.

**Figure 7:**
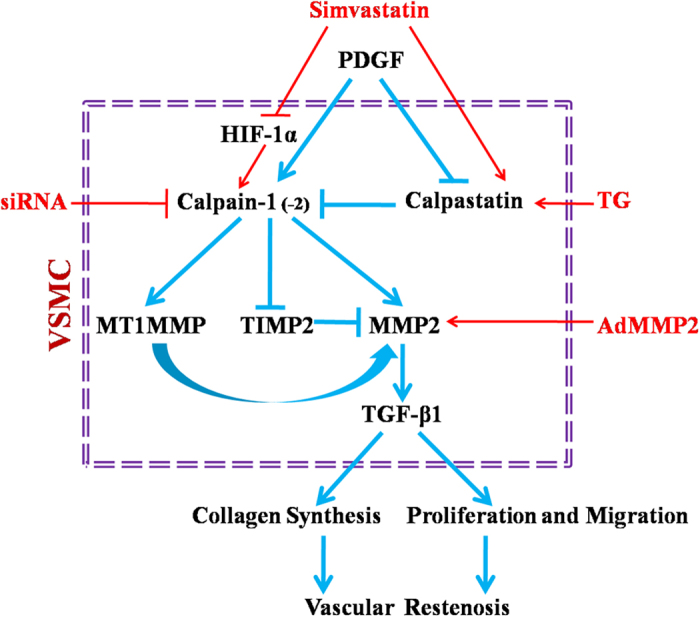
Schematic diagram depicting the protective effects of calpains inhibition in vascular restenosis by inhibition of MMP2/TGF-β1 signalling. In vascular restenosis, the functional balance of calpastatin and calpains was disturbed, leading to the activation of MMP2/TGF-β1 signalling. It is well known that the MMP2/TGF-β1 pathway is closely related to the proliferation and migration of VSMCs and collagen synthesis. Using TG mice, specific siRNAs against calpain-1/2, AdMMP2, and simvastatin, we revealed that calpastatin induction and calpains inhibition suppress the expression of MMP2/TGF-β1, subsequently preventing the proliferation and migration of VSMCs and collagen synthesis, finally attenuating vascular restenosis. Most importantly, calpain-1 is the major molecule in the development of vascular restenosis by influencing all aspects. In contrast, calpain-2 only played an auxiliary role in restenosis processes by enhancing VSMC migration. Moreover, simvastatin may inhibit the expression of calpain-1/2 by accelerating HIF-1α degradation to attenuate vascular restenosis. TG, calpastatin transgene; VSMCs, vascular smooth muscle cells; PDGF, platelet-derived growth factor; MMP2, matrix metalloproteinase 2; MT1MMP, membrane type matrix metalloproteinase-1; TIMP2, tissue inhibitor of matrix metalloproteinase-2; TGF-β1, transforming growth factor-β1. TG, calpastatin transgene; siRNA, small interfering RNA; AdMMP2, adenoviral vector carrying *MMP2*; PDGF, platelet-derived growth factor; MMP2, matrix metalloproteinase 2; MT1MMP, membrane-type matrix metalloproteinase-1; TIMP2, tissue inhibitor of matrix metalloproteinase-2; TGF-β1, transforming growth factor-β1.

In summary, we have presented data indicating that both calpastatin induction and calpains inhibition ameliorate vascular restenosis by suppression of the MMP2/TGF-β1 pathway (summarized in Fig. 7). Our findings highlight future therapeutic strategies capable of bolstering calpastatin in VSMCs to protect against in-stent restenosis for artery atherosclerotic patients.

## Additional Information

**How to cite this article**: Tang, L. *et al*. The inhibition of calpains ameliorates vascular restenosis through MMP2/TGF-β1 pathway. *Sci. Rep.* **6**, 29975; doi: 10.1038/srep29975 (2016).

## Supplementary Material

Supplementary Information
