# Supplementary material for: The inhibition of calpains ameliorates vascular restenosis through MMP2/TGF-β1 pathway
Source: Sci Rep. 2016 Jul 25;6:29975. doi: 10.1038/srep29975 (PMC4958998; doi:10.1038/srep29975)

The inhibition of calpains ameliorates vascular restenosis through MMP2/TGF-β1 pathway

Lianghu Tang^a,b,1^, MD, Haifeng Pei^a,1^, MD, PhD, Yi Yang^a,1^, MD, Xiong Wang^c^, MD,

Ting Wang^a^, MD, Erhe Gao^d^, PhD, De Li^a^, MD, Yongjian Yang^a,*^, MD, Dachun Yang^a,*^, MD, PhD

^a^ Department of Cardiology, Chengdu Military General Hospital, Chengdu 610083, China;

^b^ Department of Cardiology, The Affiliated Hospital of Southwest Medical University, Luzhou 64600, China;

^c^ Department of Cardiology, Xijing Hospital, Fourth Military Medical University, Xi’an 710032, China;

^d^ Center of Translational Medicine, Temple University School of Medicine, Philadelphia, PA 19140, USA.

^1^These authors contributed equally to this study.

**Running title:** Calpastatin and calpains in restenosis

***Address correspondence to:**

Dachun Yang, MD, PhD

Department of Cardiology

Chengdu Military General Hospital

270 Tianhui Road

Chengdu 610083, China

E-mail: [yangdachun2010@126.com](mailto:yangdachun2010@126.com)

Tel: +86-28-8657-0212

Fax: +86-28-8357-2213

**Or**

Yongjian Yang, MD, PhD

Department of Cardiology

Chengdu Military General Hospital

270 Tianhui Road

Chengdu 610083, China

E-mail: [yyj10001@126.com](mailto:yyj10001@126.com)

Tel: +86-28-8657-0211

Fax: +86-28-8357-2211

**Supplemental Figure Legends**

**Supplemental Fig. 1** Representative images of immunohistochemical staining for PCNA, MMP2, MT1MMP, and TIMP2.


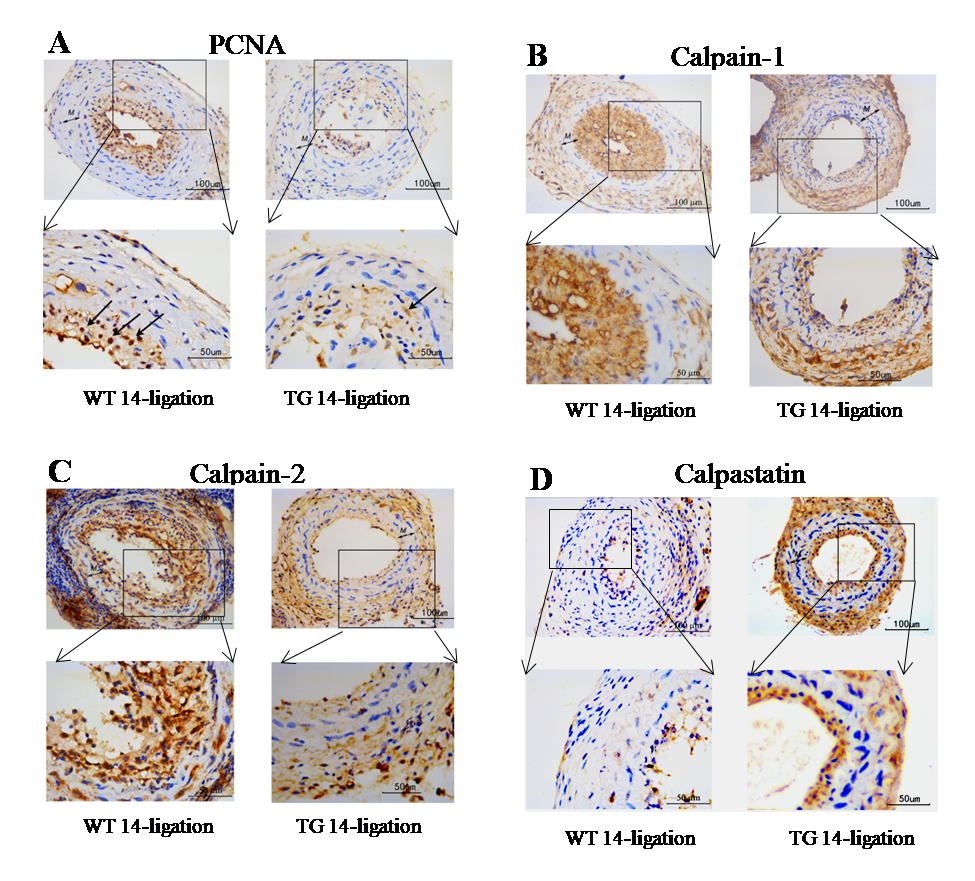


**Supplemental Fig. 2 (A)** Representative images of immunohistochemical staining for α-actin. **(B)** Representative images of cell proliferation tested by cell counting kit-8 (CCK-8) assays. **(C)** Representative images of non-directional cell migration tested by transwell migration assays. **(D)** Representative images of directional migration tested by scratch wound healing assays.


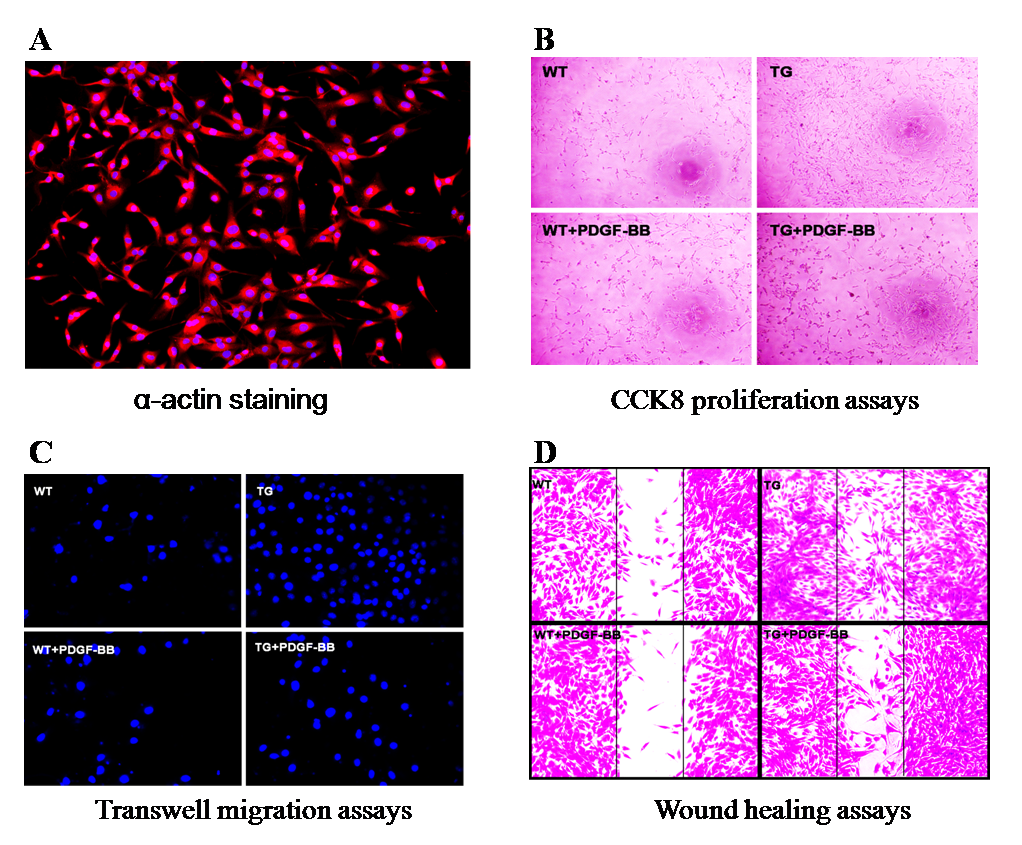

Supplement: Supplementary Information [file srep29975-s1.doc]
